# Sustained glucagon receptor antagonism in insulin-deficient high-fat-fed mice

**DOI:** 10.1530/JOE-22-0106

**Published:** 2022-08-24

**Authors:** Ryan A Lafferty, Laura M McShane, Zara J Franklin, Peter R Flatt, Finbarr P M O’Harte, Nigel Irwin

**Affiliations:** 1 Biomedical Sciences Research Institute, Centre for Diabetes, Ulster University, Coleraine, Northern Ireland, UK

**Keywords:** glucagon, glucose homeostasis, insulin sensitivity, high-fat-fed mice, streptozotocin

## Abstract

Discerning modification to the amino acid sequence of native glucagon can generate specific glucagon receptor (GCGR) antagonists, that include desHis^1^Pro^4^Glu^9^-glucagon and the acylated form desHis^1^Pro^4^Glu^9^(Lys^12^PAL)-glucagon. In the current study, we have evaluated the metabolic benefits of once-daily injection of these peptide-based GCGR antagonists for 18 days in insulin-resistant high-fat-fed (HFF) mice with streptozotocin (STZ)-induced insulin deficiency, namely HFF-STZ mice. Administration of desHis^1^Pro^4^Glu^9^-glucagon moderately (*P* < 0.05) decreased STZ-induced elevations of food intake. Body weight was not different between groups of HFF-STZ mice and both treatment interventions delayed (*P* < 0.05) the onset of hyperglycaemia. The treatments reduced (*P* < 0.05–*P* < 0.001) circulating and pancreatic glucagon, whilst desHis^1^Pro^4^Glu^9^(Lys^12^PAL)-glucagon also substantially increased (*P* < 0.001) pancreatic insulin stores. Oral glucose tolerance was appreciably improved (*P* < 0.05) by both antagonists, despite the lack of augmentation of glucose-stimulated insulin release. Interestingly, positive effects on i.p. glucose tolerance were less obvious suggesting important beneficial effects on gut function. Metabolic benefits were accompanied by decreased (*P* < 0.05–*P* < 0.01) locomotor activity and increases (*P* < 0.001) in energy expenditure and respiratory exchange ratio in both treatment groups. In addition, desHis^1^Pro^4^Glu^9^-glucagon increased (*P* < 0.01*–P* < 0.001) O_2_ consumption and CO_2_ production. Together, these data provide further evidence that peptidic GCGR antagonists are effective treatment options for obesity-driven forms of diabetes, even when accompanied by insulin deficiency.

## Introduction

It has been well established that abnormal elevation in circulating glucagon leads to an increase in hepatic glucose production and glycogen metabolism that contribute to hyperglycaemia in diabetes (Unger 1978). For this reason, blockade of glucagon receptor (GCGR) signalling has been widely regarded as a potential therapeutic option to help control blood glucose levels for the treatment of diabetes (Patil *et al.* 2020, Lafferty *et al.* 2021). In addition, some recent observations (Wang *et al.* 2021), coupled with earlier work (Okamoto *et al.* 2015, 2017), suggest that GCGR blockade can also promote recovery of functional beta-cell mass, with obvious additional benefits for diabetes. Indeed, there are several reports that GCGR knockout (KO) mice are more resistant to beta-cell destruction in response to islet stress (Conarello *et al.* 2007, Lee *et al.* 2012).

Various chemical approaches have been taken in an attempt to annul GCGR activity for therapeutic benefit, including small molecules (Mu *et al.* 2012, Guzman-Perez *et al.* 2013, Pettus *et al.* 2020), monoclonal antibodies (Kim *et al.* 2012, Okamoto *et al.* 2015, 2017) or antisense oligonucleotides (Liang *et al.* 2004, Morgan *et al.* 2019). Although all approaches possess robust glucose-lowering actions, the adverse side effect profile of each has been questioned (Patil *et al.* 2020, Lafferty *et al.* 2021). To date, it appears that peptide-based GCGR antagonists offer the best efficacy vs side effect profile (Irwin *et al.* 2013, O’Harte *et al.* 2013, Franklin *et al.* 2014, 2022, McShane *et al.* 2014). Whether this relates to the composition of the compounds in question, or overall potency and degree of GCGR blockade, remains to be determined. However, a wealth of data suggests that organic peptides, such as desHis^1^Pro^4^Glu^9^-glucagon, represent highly effective GCGR antagonists (O’Harte *et al.* 2013, Franklin *et al.* 2022). Indeed, other truncated glucagon-based peptides have recently been shown to yield selective, high potency, GCGR antagonists (Yang *et al.* 2021), supporting this as an effective approach to decrease GCGR activity. Moreover, an acylated, longer-acting, version of desHis^1^Pro^4^Glu^9^-glucagon has been described, namely desHis^1^Pro^4^Glu^9^-glucagon(Lys^12^PAL), that also effectively antagonises the GCGR (Franklin *et al.* 2014). This analogue has a palmitic acid covalently attached to the Lys^12^ residue of desHis^1^Pro^4^Glu^9^-glucagon via a γ-glutamyl spacer molecule, delivering a significantly extended pharmacodynamic profile (O’Harte *et al.* 2013). Notably, our previous work fully characterises the *in vitro* and acute *in vivo* biological action profile of both desHis^1^Pro^4^Glu^9^-glucagon and desHis^1^Pro^4^Glu^9^-glucagon(Lys^12^PAL), including effects on cAMP accumulation, insulin secretion, inhibition of glucagon action, glucose disposal and islet hormone secretion (O’Harte *et al.* 2013, Franklin *et al.* 2014).

Moreover, we have also previously shown that sustained administration of desHis^1^Pro^4^Glu^9^-glucagon, or its Lys^12^ acylated counterpart, can reverse aspects of genetically induced and dietary-induced obesity-related diabetes in obese-diabetic (*ob/ob*) and high-fat-fed (HFF) mice, respectively (O’Harte *et al.* 2014). However, both these murine models of diabetes are associated with adaptive beta-cell expansion prior to the development of overt diabetes. In this regard, administration of the beta-cell toxin, streptozotocin (STZ), can counter beta-cell compensation and prevent such innate adaptations (Furman 2015). Thus, HFF mice with STZ-induced compromised beta-cells are characterised by obstruction of the classical beta-cell hypertrophy in response to prolonged high-fat feeding (Tanday *et al.* 2021). Therefore, this HFF-STZ murine model represents an ideal tool to fully explore the positive effects of peptide-based GCGR antagonists in obesity-driven forms of diabetes, where restoration of functional beta-cell mass would be highly advantageous. Notably, the benefits of GCGR blockade are believed to require at least some residual beta-cell function (Damond *et al.* 2016), which would be the case for HFF-STZ mice (Tanday *et al.* 2021).

Consequently, in the current study, we have investigated the impact of once-daily treatment with desHis^1^Pro^4^Glu^9^-glucagon or desHis^1^Pro^4^Glu^9^-glucagon(Lys^12^PAL) in HFF-STZ mice for 18 days. Effects on food and fluid intake as well as body weight and circulating glucose were assessed at regular intervals. The metabolic status of the mice was then examined at the end of the treatment period through glucose and insulin tolerance tests. Finally, aspects of indirect calorimetry and pancreatic hormone content were also investigated. Taken together, we reveal that peptidic GCGR antagonists possess metabolic benefits following STZ-induced beta-cell insult in insulin-resistant HFF mice, which merits further investigation in terms of translation to the clinical setting.

## Materials and methods

### Peptides

All peptides were synthesised by Synpeptide (Shanghai, China) at 95% purity, which was confirmed in-house by high-performance liquid chromatography (HPLC) and matrix-assisted laser desorption/ionisation-time of flight (MALDI-TOF) mass spectrometry (MS), as previously described (Lafferty *et al.* 2020).

### Animals

Young male NIH Swiss mice (10-week-old; *n*  = 8) were maintained on high-fat diet (45% fat, 20% protein and 25% carbohydrates; percent of total energy of 26.15 kJ/g; Special Diets Services, Witham, Essex, UK) for 12 weeks, by which stage obesity was clearly manifested. After this period, mice were administered with a single large i.p. dose of STZ (4-h fast, 125 mg/kg bw, dissolved in sodium citrate buffer, pH 4.5). A separate group of HFF mice that did not receive STZ injection were employed as an additional control group. Appropriate numbers of non-diabetic control mice were not available for inclusion in the current study, but the basic phenotypes of HFF mice such as obesity, impaired, glucose tolerance, hyperinsulinaemia and insulin resistance were confirmed.

### Chronic *in vivo* experiments

Groups (*n* = 8) of HFF-STZ mice received once-daily i.p. injections (10:00 h) of saline vehicle (0.9% (w/v) NaCl), desHis^1^Pro^4^Glu^9^-glucagon or desHis^1^Pro^4^Glu^9^-glucagon(Lys^12^PAL) (both at 25 nmol/kg bw) for 18 days, starting on the same day that STZ was administered. To acclimatise mice to the injection regimen, all mice received once-daily i.p. injections of saline over a 6-day run in period. Mice were maintained on high-fat diet (45%) throughout the experiment. At regular intervals, cumulative energy and fluid intake, body weight and non-fasting circulating glucose were assessed. At the end of the treatment period, oral and i.p. glucose tolerance (18 mmol/kg bw; i.p. or oral as appropriate; 18-h fasted) as well as insulin sensitivity (5 U/kg bovine insulin; i.p.; non-fasted) tests were conducted. Aspects of indirect calorimetry were measured using an Oxymax Comprehensive Laboratory Animal Monitoring System (CLAMS), with 18 h acclimation prior to recordings (Columbus Instruments, Columbus, OH, USA). Following the acclimatisation period, O_2_ consumption, CO_2_ production, respiratory exchange ratio (RER), energy expenditure and locomotor activity were assessed, as described previously (O’Harte *et al.* 2018). All animal experiments were approved by Ulster University Animal Ethics Review Committee and conducted in accordance with the UK Animals (Scientific Procedures) Act 1986.

### Biochemical analyses

Blood samples were obtained from conscious mice via the cut tip on the tail vein, and blood glucose was immediately measured using an Ascencia Contour blood glucose meter (Bayer Healthcare, Newbury, UK). Pancreatic or plasma insulin and glucagon, as appropriate, were measured by in-house RIA (Flatt & Bailey 1981) or commercially available ELISA (EZGLU-30K, Merck Millipore), respectively.

### Statistical analyses

Statistical tests were conducted using GraphPad PRISM software (Version 5.0). Values are expressed as mean ± s.e.m. Comparative analyses between groups were performed using a one-way or two-way ANOVA with Bonferroni’s *post hoc* test, as appropriate. Differences were deemed significant if *P* < 0.05.

## Results

### Effects of desHis^1^Pro^4^Glu^9^-glucagon or desHis^1^Pro^4^Glu^9^-glucagon(Lys^12^PAL) on food and fluid intake, body weight, circulating glucose and glucagon in HFF-STZ mice

**Figure 1:**
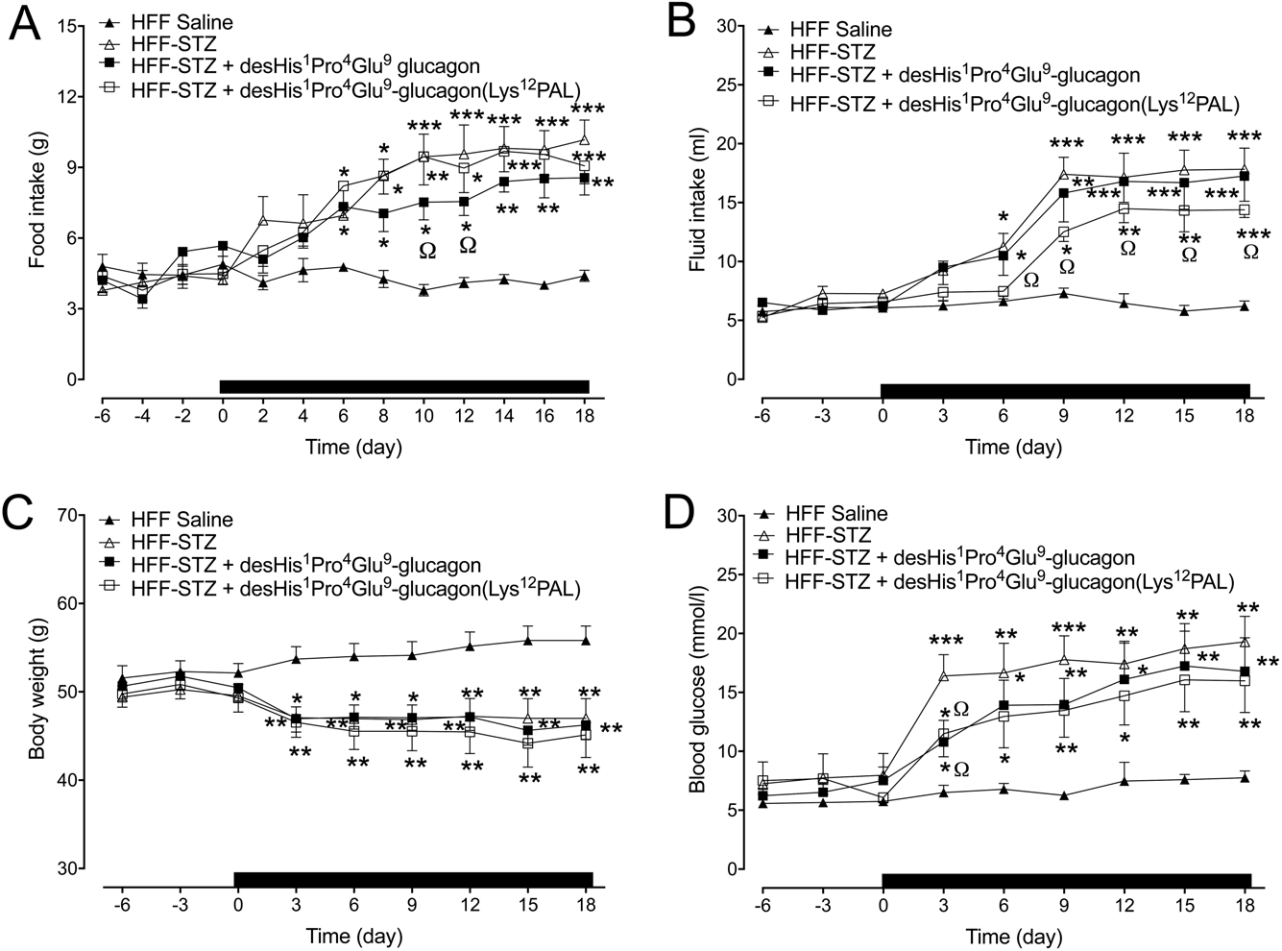
Effects of once-daily administration of desHis^1^Pro^4^Glu^9^-glucagon or desHis^1^Pro^4^Glu^9^-glucagon (Lys^12^PAL) (each at 25 nmol/kg bw) for 18 days on cumulative food intake (A), cumulative fluid intake (B), body weight (C) and blood glucose (D) in HFF-STZ mice. Measurements were taken 6 days prior to and throughout the treatment period, at regular intervals. The treatment period is highlighted by the horizontal black bar parallel to x-axis. Values are mean ± s.e.m. (*n* = 8). *P < 0.05, **P < 0.01, ***P < 0.001 compared to HFF-STZ saline controls. ^Ω^*P* < 0.05 compared to HFF saline controls.

Food intake was significantly (*P* < 0.05*–P* < 0.001) increased in all HFF mice that received STZ injection (Fig. 1A). Only treatment with desHis^1^Pro^4^Glu^9^-glucagon led to reductions (*P* < 0.05) of STZ-induced elevations of food intake that was evident on days 10 and 12 (Fig. 1A). Interestingly, STZ-related increases (*P* < 0.05*–P* < 0.001) in fluid intake were partially reversed (*P* < 0.05) in desHis^1^Pro^4^Glu^9^-glucagon(Lys^12^PAL)-treated mice, but not by desHis^1^Pro^4^Glu^9^-glucagon (Fig. 1B). Body weight was reduced in all STZ mice, with treatment interventions having no impact on this parameter (Fig. 1C). As expected, STZ administration resulted a significant (*P* < 0.001) sustained increase in blood glucose levels from day 3 onwards (Fig. 1D). Both desHis^1^Pro^4^Glu^9^-glucagon and desHis^1^Pro^4^Glu^9^-glucagon(Lys^12^PAL) partially protected (*P* < 0.05) against STZ-induced elevations of glucose, but these mice still had increased (*P* < 0.05–*P* < 0.01) circulating glucose when compared to HFF control mice (Fig. 1D). In terms of circulating glucagon concentrations, STZ treatment increased (*P* < 0.001) circulating glucagon levels in HFF mice on day 18 when compared to lean controls (51.2 ± 13.6 vs 24.3 ± 8.8 pg/mL, respectively), but this effect was fully reversed by both treatment regimens where circulating glucagon was between 19.8 and 23.4 ± 6.7 pg/mL in these mice on day 18.

### Effects of desHis^1^Pro^4^Glu^9^-glucagon or desHis^1^Pro^4^Glu^9^-glucagon(Lys^12^PAL) on glucose tolerance and insulin sensitivity in HFF-STZ mice

**Figure 2:**
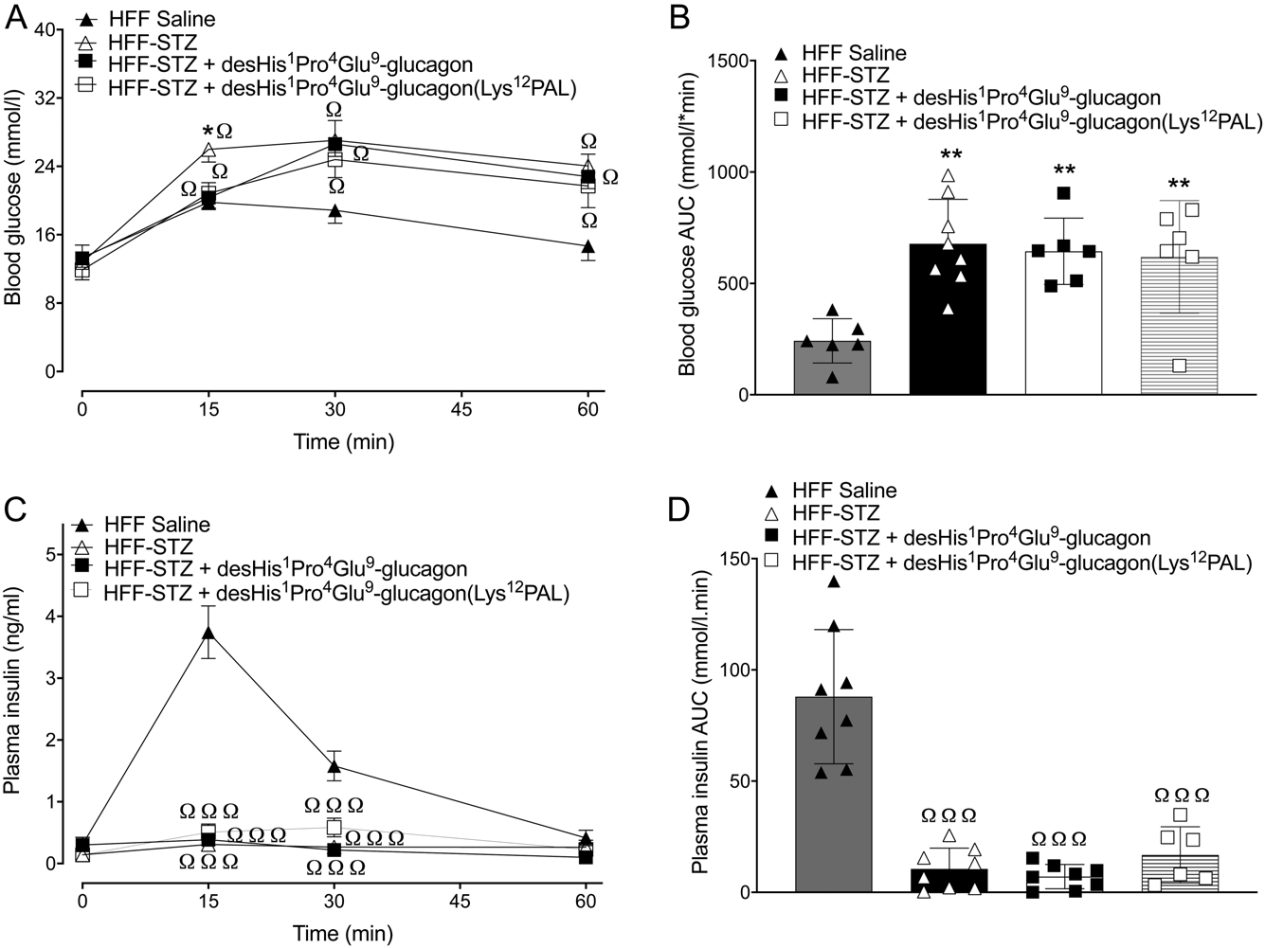
Effects of once-daily administration of desHis^1^Pro^4^Glu^9^-glucagon or desHis^1^Pro^4^Glu^9^-glucagon(Lys^12^PAL) (each at 25 nmol/kg bw) for 18 days on i.p. glucose tolerance in HFF-STZ mice (18 mmol/kg bw). Blood glucose (A) and associated plasma insulin responses (C) with respective areas under the curves (B and D) are provided. Values are mean ± s.e.m. (*n* = 8). *P < 0.05 compared to HFF-STZ saline controls. ^Ω^*P* < 0.05, ^ΩΩΩ^*P* < 0.001 compared to HFF saline controls.

**Figure 3:**
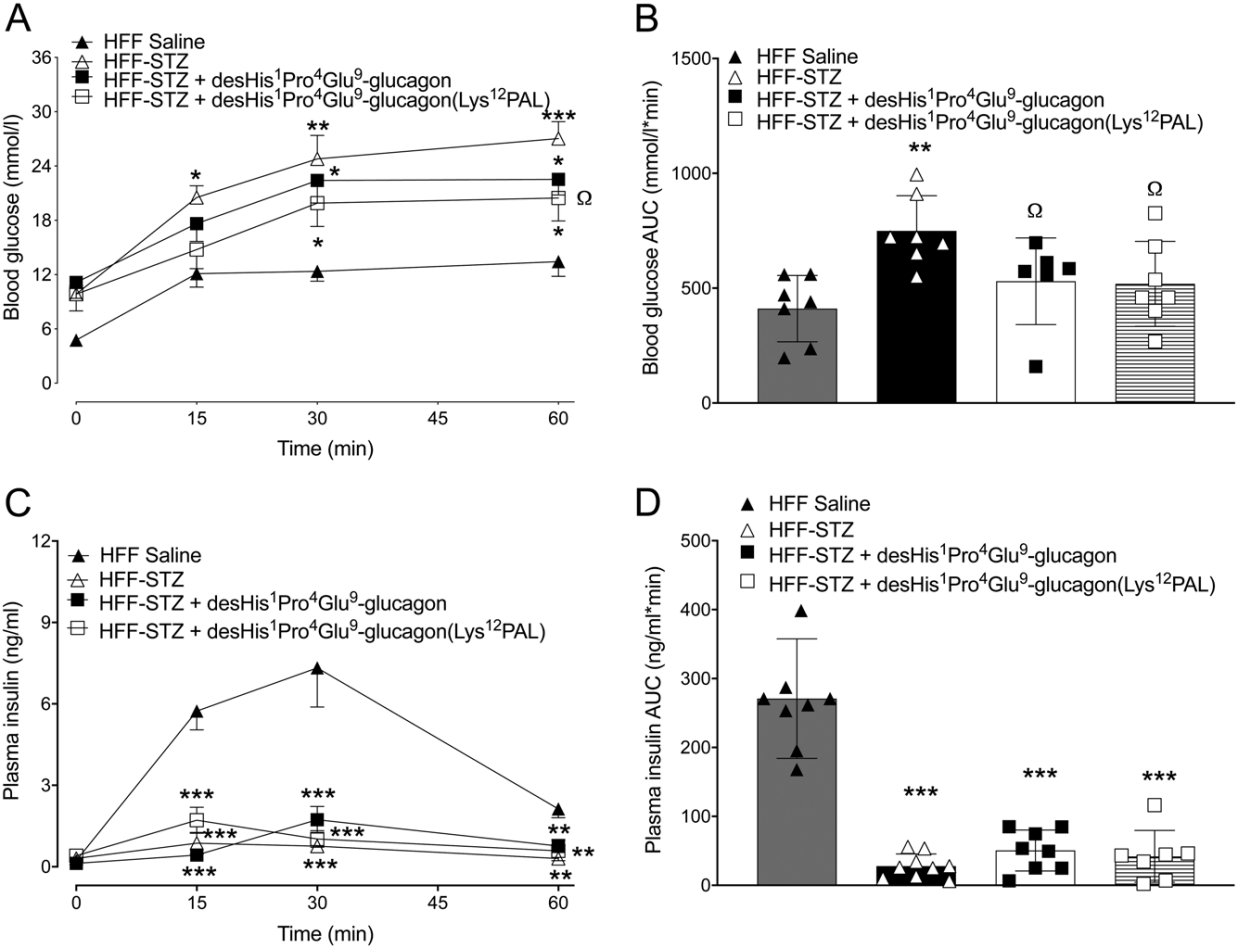
Effects of once-daily administration of desHis^1^Pro^4^Glu^9^-glucagon or desHis^1^Pro^4^Glu^9^-glucagon(Lys^12^PAL) (each at 25 nmol/kg bw) for 18 days on oral glucose tolerance in HFF-STZ mice (18 mmol/kg bw). Blood glucose (A) and associated plasma insulin responses (C) with respective areas under the curves (B and D) are provided. Values are mean ± s.e.m. (*n* = 8). *P < 0.05 compared to HFF-STZ saline controls. ^Ω^ *P* < 0.05 compared to HFF saline controls.

**Figure 4:**
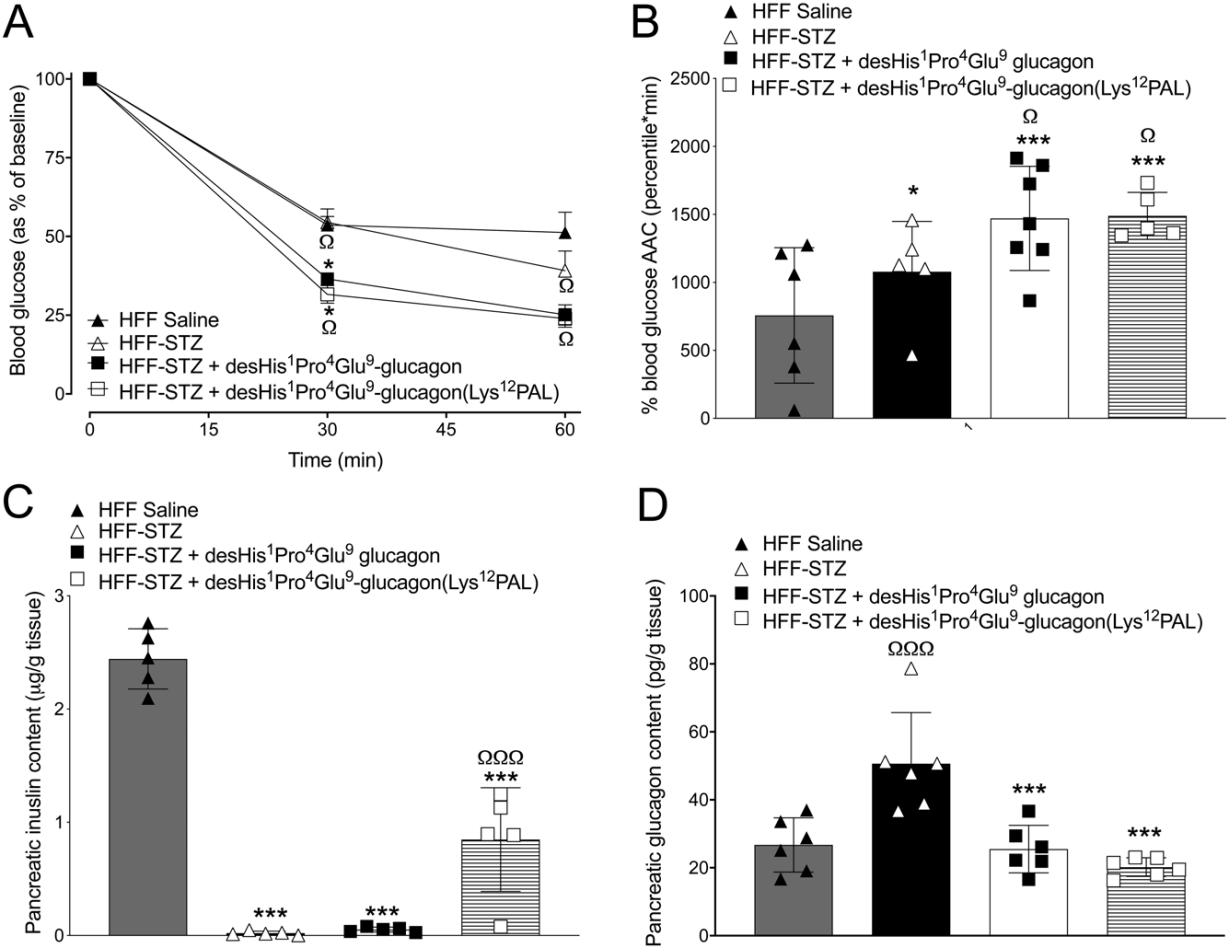
Effects of once-daily administration of desHis^1^Pro^4^Glu^9^-glucagon or desHis^1^Pro^4^Glu^9^-glucagon(Lys^12^PAL) (each at 25 nmol/kg bw) for 18 days on insulin sensitivity (A) in HFF-STZ mice, with the related area above the curve (B) presented. Insulin was administered by i.p. injection at 5 IU/kg/bw in non-fasted mice. Additionally, effects on pancreatic hormone content were assessed on day 18. Pancreatic glucagon (C) and insulin (D) levels were assessed in excised pancreatic tissue via a commercially available ELISA or in-house RIA, respectively. Values are mean ± s.e.m. (*n* = 8). *P < 0.05, ***P < 0.001 compared to HFF-STZ saline controls. ^Ω^*P* < 0.05, ^ΩΩΩ^*P* < 0.001 compared to HFF saline controls.

Following an i.p. glucose challenge, glucose levels were significantly lower (*P* < 0.05) 15 min post injection in both desHis^1^Pro^4^Glu^9^-glucagon and desHis^1^Pro^4^Glu^9^-glucagon(Lys^12^PAL)-treated HFF-STZ mice when compared to saline controls (Fig. 2A). However, this reduction was not sustained at 30 and 60 min (Fig. 2A), and there was no difference in 0–60 min glucose AUC values between all HFF-STZ groups of mice (Fig. 2B). Glucose-induced insulin secretory responses were almost absent in all HFF-STZ mice, with only control HFF mice displaying any real glucose-induced elevations of insulin concentrations (Fig. 2C and D). The benefits of desHis^1^Pro^4^Glu^9^-glucagon and desHis^1^Pro^4^Glu^9^-glucagon(Lys^12^PAL) treatment were more prominent following an oral glucose tolerance challenge (Fig. 3A and B). Thus, although individual glucose levels were still elevated in the treatment groups compared to HFF controls (Fig. 3A), 0–60 min AUC values were decreased (*P* < 0.05) by desHis^1^Pro^4^Glu^9^-glucagon and desHis^1^Pro^4^Glu^9^-glucagon(Lys^12^PAL) when compared to STZ-diabetic control mice, and not significantly different from HFF controls (Fig. 3B). However, glucose-induced insulin concentrations were not noticeably amplified by either treatment (Fig. 3C and D). In some agreement with this, the hypoglycaemic action of exogenous insulin was significantly (*P* < 0.05–*P* < 0.001) augmented by desHis^1^Pro^4^Glu^9^-glucagon and desHis^1^Pro^4^Glu^9^-glucagon(Lys^12^PAL), when compared to HFF-STZ or HFF control mice (Fig. 4A and B). Interestingly, STZ administration alone also appeared to enhance (*P* < 0.05) peripheral insulin action in HFF mice (Fig. 4A and B). As anticipated, administration of STZ significantly (*P* < 0.001) depressed pancreatic insulin content, but 18 days therapy with desHis^1^Pro^4^Glu^9^-glucagon(Lys^12^PAL) was able to partially reverse (*P* < 0.001) this detrimental effect (Fig. 4C). STZ also increased (*P* < 0.001) pancreatic glucagon content of HFF mice, but this effect was fully reversed by both desHis^1^Pro^4^Glu^9^-glucagon and desHis^1^Pro^4^Glu^9^-glucagon(Lys^12^PAL) treatment (Fig. 4D).

### Effects of desHis^1^Pro^4^Glu^9^-glucagon or desHis^1^Pro^4^Glu^9^-glucagon(Lys^12^PAL) on indirect calorimetry and locomotor activity in HFF-STZ mice

**Figure 5:**
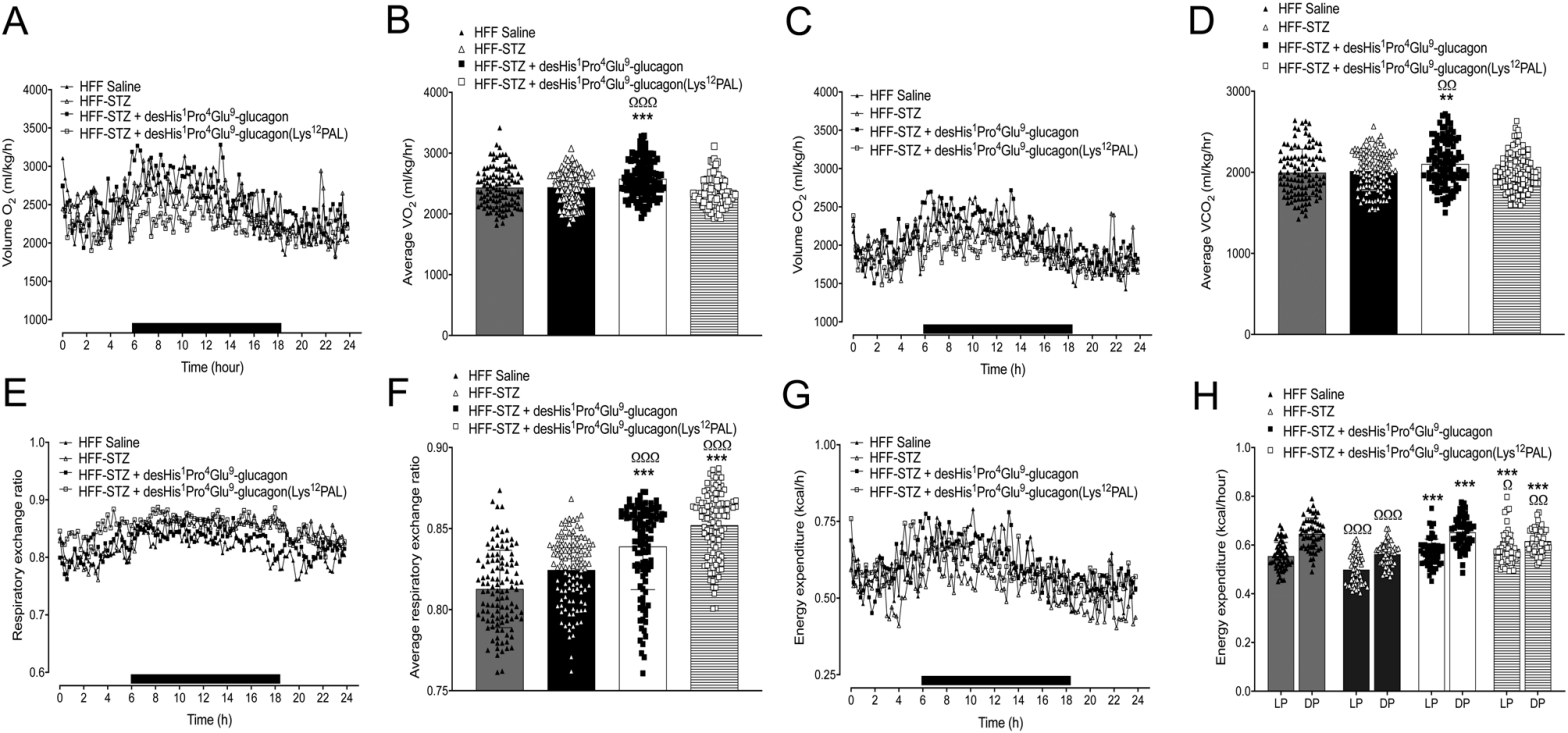
Effects of once-daily administration of desHis^1^Pro^4^Glu^9^-glucagon or desHis^1^Pro^4^Glu^9^-glucagon(Lys^12^PAL) (each at 25 nmol/kg bw) for 18 days on O_2_ consumption (A and B), CO_2_ production (C and D), respiratory exchange ratio (RER) (E and F) and energy expenditure (G and H) in HFF-STZ mice. Mice were placed in CLAMS metabolic chambers for 18 h to acclimatise, and measurements were obtained over a further 24 h period (12 h dark period as shown by black bar parallel to x-axis) at the end of the treatment period. O_2_ consumption and CO_2_ production were measured for 30 s at 25 min intervals (A and C). RER was calculated by dividing VCO_2_ by VO_2_ (E and F). Energy expenditure was calculated using RER with the following equation: (3.815 + 1.232 × RER) × VO_2_ (G). Average energy expenditure is also provided (H), separated into the light (LP) and dark phases (DP). Overall incremental data are presented in panels B, D, F and G, where each data point represents information collected at individual time-points over the 24 h period. Values are mean ± s.e.m. (*n* = 6). **P < 0.01, ***P < 0.001 compared to HFF-STZ saline controls. ^Ω^*P* < 0.05, ^ΩΩ^*P* < 0.01, ^ΩΩΩ^*P* < 0.001 compared to HFF saline controls.

**Figure 6:**
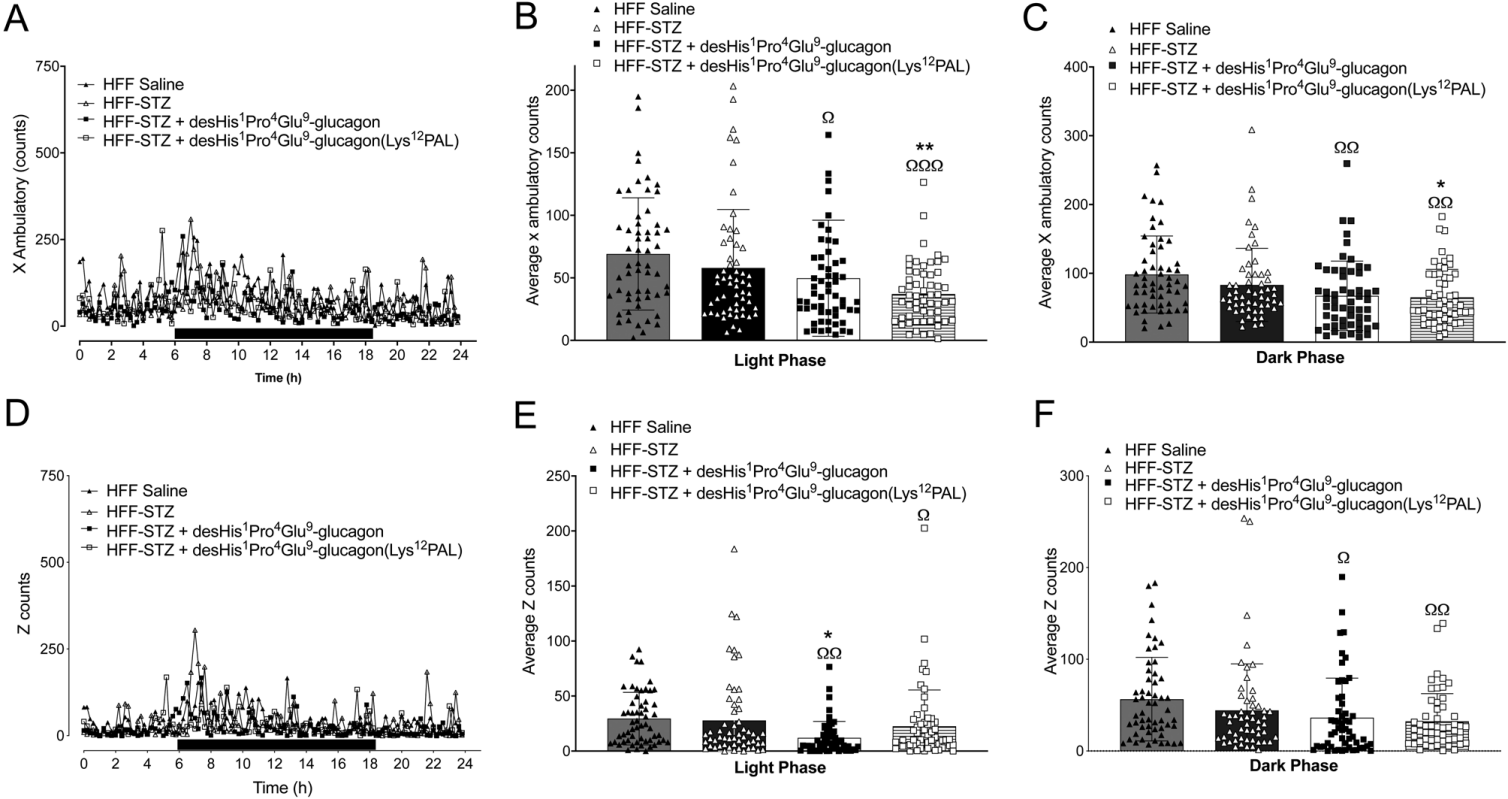
Effects of once-daily administration of desHis^1^Pro^4^Glu^9^-glucagon or desHis^1^Pro^4^Glu^9^-glucagon(Lys^12^PAL) (each at 25 nmol/kg bw) for 18 days on locomotor activity in HFF-STZ mice. Mice were placed in CLAMS metabolic chambers for 18 h to acclimatise, and measurements were obtained over a further 24 h period (12 h dark period as shown by black bar parallel to x-axis) at the end of the treatment period. Activity counts on x-axis (A, B and C) and z-axis (D, E and F) were recorded at 60-s intervals. Overall incremental data are presented in panels B, C, E and F, where each data point represents information collected at individual time-points over the 24 h period. Values are mean ± s.e.m. (*n* = 6). *P < 0.05, **P < 0.01 compared to HFF-STZ saline controls. ^Ω^*P* < 0.05, ^ΩΩ^*P* < 0.01, ^ΩΩΩ^*P* < 0.001 compared to HFF saline controls.

Consumption of O_2_ was similar in HFF and HFF-STZ mice on day 18, but desHis^1^Pro^4^Glu^9^-glucagon increased (*P* < 0.001) this parameter (Fig. 5A and B). Consistent with these findings, desHis^1^Pro^4^Glu^9^glucagon also increased CO_2_ production (*P* < 0.01) in comparison to both HFF and HFF-STZ control mice (Fig. 5C and D). In addition, desHis^1^Pro^4^Glu^9^-glucagon and desHis^1^Pro^4^Glu^9^-glucagon(Lys^12^PAL) treatment resulted in a significant (*P* < 0.001) increase in RER (Fig. 5E and F). Energy expenditure was decreased (*P* < 0.001) by STZ administration in HFF mice, which was fully reversed by both GCGR antagonists (Fig. 5G and H). Interestingly, both treatment interventions decreased (*P* < 0.05–*P* < 0.01) X beam ambulatory breaks vs HFF-STZ controls during both the light and dark phases (Fig. 6A, B and C). A similar effect of desHis^1^Pro^4^Glu^9^-glucagon was also noted in terms of Z-beam breaks during the light phase (*P* < 0.05), which represent vertical activity levels such as mouse rearing events (Fig. 6D, E and F). Both peptide treatments had significantly (*P* < 0.05–*P* < 0.001) reduced X and Z beam breaks when compared to saline-treated HFF controls (Fig. 6A, B, C, D, E and F).

## Discussion

The interplay between pancreatic alpha- and beta-cell signalling is intriguing, with secretions from beta-cells directly inhibiting alpha-cell function, whilst alpha-cells release factors that are stimulatory for beta-cells (English & Irwin 2019, Moede *et al.* 2020). Coupled with recent awareness that mature beta-cells de-differentiate into alpha-cells (Weir *et al.* 2013) and that alpha-cells can act as progenitors for functional beta-cells (Habener & Stanojevic 2012), modulation of alpha-cell activity could hold promise for the treatment of diabetes. Indeed, alpha-to-beta-cell lineage conversion is enhanced in GCGR KO mice (Damond *et al.* 2016), and more recently, human alpha-cells were shown to be capable of reprogramming into glucose-sensitive insulin-secreting cells to help ameliorate diabetes in mice (Furuyama *et al.* 2019). In this regard, inhibition of GCGR signalling has long been considered as a potential means of effectively controlling blood glucose levels (Unger 1978).

In the current study, we employed a high single dose of STZ as an established method to induce beta-cell cytotoxicity and perturb insulin secretory function in HFF mice (Deeds *et al.* 2011, Millar *et al.* 2017). Thus, HFF mice are classically characterised by the manifestation of insulin resistance leading to subsequent compensatory beta-cell expansion and hyperinsulinaemia (Ahrén *et al.* 2010). Notably, saline-treated control HFF mice did not present with overt hyperglycaemia, but as expected, glucose intolerance was evident following a glucose challenge likely as a result of dietary-induced insulin resistance (Ahrén *et al.* 2010). However, hyperglycaemia was clearly apparent in all GCGR antagonist-treated HFF-STZ mice by day 18, treatment intervention appeared to delay onset. Indeed, the acylated GCGR analogue partially restored pancreatic insulin concentrations, which may be linked to the more protracted bioactive profile of desHis^1^Pro^4^Glu^9^-glucagon(Lys^12^PAL) over desHis^1^Pro^4^Glu^9^-glucagon (Franklin *et al.* 2014). In agreement, GCGR blockade has recently been demonstrated to promote recovery of functional beta-cell mass in diabetic mice (Wang *et al.* 2021). Regrettably, a technical issue during tissue processing thwarted our efforts to investigate aspects of pancreatic islet morphology, including beta-cell mass and turnover, that would help to validate our observations. Interestingly, the augmented pancreatic insulin content, which was particularly apparent in desHis^1^Pro^4^Glu^9^-glucagon(Lys^12^PAL)-treated HFF-STZ mice, was not matched by prominent improvements in insulin secretory responses or glucose levels. Thus, further investigation of beta-cell secretory function and responsiveness would be required to uncover the relationship between increased insulin stores and translation to more obvious improvements of metabolism in these mice, although improvements in insulin action might also be important in this regard. Circulating and pancreatic glucagon levels were reduced in all GCGR antagonist-treated HFF-STZ, which contrasts with observations using small molecule GCGR inhibitors (Mu *et al.* 2011, 2012), but complements previous work with peptidic GCGR antagonists (Franklin *et al.* 2014, McShane *et al.* 2014, O’Harte *et al.* 2014). This also highlights the improved adverse effect profile of peptidic GCGR antagonists over other methods employed to inhibit GCGR signalling. As such, rebound hyperglycaemia has been observed on treatment termination with some small molecule GCGR antagonists (Sloop *et al.* 2004), likely because of their actions to elevate circulating glucagon.

Benefits on glucose tolerance were more apparent following an oral as opposed to i.p. glucose challenge in desHis^1^Pro^4^Glu^9^-glucagon and desHis^1^Pro^4^Glu^9^-glucagon(Lys^12^PAL)-treated HFF-STZ mice. In accordance with this, GCGR antagonism-mediated improvements in glycaemic control have been suggested to be dependent on functional GLP-1 receptors (Gu *et al.* 2010). Indeed, more recent studies have demonstrated that GCGR blockade can promote intestinal L-cell proliferation (Lang *et al.* 2020*a*) and inhibit L-cell apoptosis (Lang *et al.* 2020*b*), leading to elevated GLP-1 synthesis and secretion. In agreement, inhibition of the incretin hormone degrading enzyme, dipeptidyl peptidase-4, improves the effectiveness of GCGR inhibition in diabetic mice (Mu *et al.* 2011). It has also been suggested that combined GLP-1 receptor activation and GCGR inhibition possesses beneficial actions (Claus *et al.* 2007). Unfortunately, we were unable to measure circulating GLP-1 concentrations in the current study due to the limited volume of blood that can be withdrawn from mice. However, we have recently shown that combined administration of a peptidic GCGR antagonist, with the well-characterised GLP-1 receptor mimetic exendin-4, exerts limited additive metabolic benefits (Franklin *et al.* 2022). Thus, activation of receptors for the sister incretin hormone of GLP-1, namely glucose-dependent insulinotropic polypeptide, may offer a more attractive paradigm in terms of combination therapy with GCGR antagonism (McShane *et al.* 2016). However, intestinal L-cell number has also been demonstrated to be reduced by STZ administration (Vasu *et al.* 2015), which could represent another confounding factor in our current observations. Thus, both pancreatic beta-cells and enteroendocrine L-cells appear to have limited antioxidant defence mechanisms (Lenzen 2008, Vasu *et al.* 2015). Although, in this respect, it should be noted that by their very nature, intestinal mucosal cell turnover is rapid, with efficient cellular replacement by differentiating stem cells that arise from intestinal crypts (Roth & Gordon 1990, Schonhoff *et al.* 2004).

Of note is the improvement of glucose handling in the absence of any real augmentation of insulin concentrations, this being despite elevated pancreatic insulin content in desHis^1^Pro^4^Glu^9^-glucagon(Lys^12^PAL)-treated HFF-STZ mice. It follows that insulin action must be enhanced in these mice, which was indeed apparent following exogenous insulin injection. Similar observations have been made previously following STZ treatment in GCGR KO mice (Lee *et al.* 2011). In the absence of GCGR signalling, hepatic glucose output and the positive effects of GCGR signalling on basal metabolic rate are also likely to be much reduced (Breton *et al.* 1983). In good accord with this, in the current study, the peptidic GCGR antagonists both decreased physical activity in HFF-STZ mice. However, the ability of desHis^1^Pro^4^Glu^9^-glucagon(Lys^12^PAL), and particularly desHis^1^Pro^4^Glu^9^-glucagon, to increase energy expenditure does contrast with this notion, but this may simply highlight the plasticity of signalling pathways involved in energy homeostasis (Smith *et al.* 2018). The slight difference in efficacy between desHis^1^Pro^4^Glu^9^-glucagon and desHis^1^Pro^4^Glu^9^-glucagon(Lys^12^PAL) in terms of indirect calorimetry data could be related to free vs albumin-bound drug, where it is often considered that albumin binding reduces bioactivity of peptides (Miyakawa *et al.* 2013). However, more detailed pharmacokinetic studies, that are outside the scope of the current investigation, would be required to confirm this. In a similar fashion, there were also slight differences between the effect of both peptides on food and fluid intake. Thus, desHis^1^Pro^4^Glu^9^-glucagon had a mild and transient impact on moderating STZ-induced elevations of feeding, whereas desHis^1^Pro^4^Glu^9^-glucagon(Lys^12^PAL) exerted more enduring effects to counter increased fluid intake in HFF-STZ mice. Although peptide pharmacodynamic profiles may also be important in this observation, we are unable to discount alterations in the passage of either peptide through the blood–brain barrier and the subsequent impact on hypothalamic circuits that regulate energy intake and thirst (Woods 2013).

It is established that glucagon plays an important role in lipid oxidation and metabolism (Galsgaard *et al.* 2019), and our observed increases in RER evoked by sustained GCGR antagonism likely partly reflects this. Thus, carbohydrate oxidation drives RER to a value closer to 1.0, with fatty acid oxidation reducing this to 0.7 (Rosenkilde *et al.* 2010, Purdom *et al.* 2018). Hyperaminoacidaemia has also been reported following inhibition of GCGR signalling and assessment of plasma amino acid levels would have been interesting in this regard (Richter *et al.* 2022). The impact of the high-fat background diet (45%), enduring insulin deficiency and small GCGR antagonist-induced changes in food intake and body weight need to be considered in terms of overall effects on carbohydrate metabolism. In that respect, GCGR KO mice are reported to be resistant to high-fat feeding-induced obesity (Conarello *et al.* 2007), but the possibility for life-long adaptations in these animals should not be overlooked. However, differences in the magnitude of GCGR signalling annulment between genetic and chemical receptor blockade could also be a factor. Thus, similar to the current setting, prolonged treatment with a small molecule GCGR antagonist did not affect body weight in HFF mice (Mu *et al.* 2011), this being despite increased energy expenditure with desHis^1^Pro^4^Glu^9^-glucagon and desHis^1^Pro^4^Glu^9^-glucagon(Lys^12^PAL) therapy. The current treatment regimen entailed once-daily peptide treatment for 18 days and whether extended dosing periods would lead to more discernible benefits on metabolism in HFF-STZ mice still needs to be established.

In summary, the current study establishes that peptide-based GCGR antagonism exerts notable benefits in obesity-driven forms of diabetes, even in the presence of insulin deficiency. As well as delaying the onset of hyperglycaemia, desHis^1^Pro^4^Glu^9^-glucagon, and particularly desHis^1^Pro^4^Glu^9^-glucagon(Lys^12^PAL), improved glucose handling and insulin action in addition to augmenting pancreatic insulin stores. Our observations further support the promise of peptidic GCGR antagonists as a new class of drugs for the management of various forms of diabetes.

## Declaration of interest

The authors declare that there is no conflict of interest that could be perceived as prejudicing the impartiality of the research reported.

## Funding

This work was supported by an Invest Northern Ireland Proof-of-Concept grant (PoC106), a Department for the Economy, Northern Ireland PhD studentship and Ulster University Selective Research Funding

## Data availability statement

The authors declare that the data supporting the findings of this study are available within the article. Any additional raw data supporting the conclusions of this article will be made available by the authors, without undue reservation.

## Author contribution statement

N I and F O H conceived/designed and supervised the study. N I, R A L and P R F drafted the manuscript. L M McS and Z J F and participated in the conduct/data collection and analysis and interpretation of data. All authors revised the manuscript critically for intellectual content and approved the final version of the manuscript.
